# 

**Published:** 1890-12

**Authors:** 


### CONTENTS.

*EDITORIALS.* PAGE

The Dose, . . . . . . . . . . . . . . . . 529
Grafts, . . . . . . . . . . . . . . . . . 532
Dr. Dudgeon on High Potencies, . . . . . . . . 533
The Other Side, . . . . . . . . . . . . . . 535
Dr. Samuel Lilienthal, . . . . . . . . . . . 536
The Teaching of Pure Homœopathy in the Colleges, . . . 537

*MISCELLANEOUS.*

Another Dose About the Dose. S. L., . . . . . . . 538
Displacements of the Uterus. (Proceedings of I. H. A. Morning Session, June 25th, 1890), . . . . . . . . . . 540
Gonorrhœa and Homœopathy. T. F. Allen, M. D., . . . . . 548
"Nuggets." C. Carleton Smith, M. D., . . . . . . . 549
British Medicinal Plants. Alfred Heath, M. D., F. L. S., . . 553
Our Daily Experience. Wm. Steinrauff, M. D., . . . . . 557
Some Verifications. Ella M. Tuttle, M. D., . . . . . . 558
Dr. J. C. Boardman's Provings and Clinical Cases. E. W. Berridge, M. D., . . . . . . . . . . . . . . 559

*BOOK NOTICES.*

Ointments and Oleates Especially in Diseases of the Skin, . . 562
A Repertory of Convulsions, . . . . . . . . . . 563
The Argonaut, . . . . . . . . . . . . . . 563
Transactions of the Maine Homœopathic Medical Society, . . 563
The People's Health Journal of Chicago, . . . . . . . 563
Census Bulletin, No. 12, . . . . . . . . . . . 564
Chemical Lecture Notes, . . . . . . . . . . . 564

*NOTES AND NOTICES.*

Removals, . . . . . . . . . . . . . . . . 564

